# Medical genomics at Belyaev Conference – 2017

**DOI:** 10.1186/s12920-018-0324-3

**Published:** 2018-02-13

**Authors:** Yuriy L. Orlov, Julio R. Fernandez-Masso, Ming Chen, Ancha V. Baranova

**Affiliations:** 1 Institute of Cytology and Genetics SB RAS, Novosibirsk, Russia; 2 Novosibirsk State University, Novosibirsk, Russia; 3 Centre of Genetic Engineering and Biotechnology, Havana, Cuba; 4 Zhejiang University, Hangzhou, China; 5 Research Centre of Medical Genetics, Moscow, Russia; 6 George Mason University, Fairfax, VA USA

Current collection continues the series of BioMed Central special post-conference issues presenting the highlights from the set of meetings on bioinformatics and systems biology held in Novosibirsk and Moscow, Russia in 2017.

Year 2017 marks the 100-th anniversary since birth of Professor Dmitry K. Belyaev (1917–1985), Full Member of the USSR Academy of Sciences, world-famous visionary in evolution and genetics. In view of this memorable date, the Institute of Cytology and Genetics of the Siberian Branch of the Russian Academy of Sciences (ICG SB RAS) held international Belyaev Conference on Genetics (Novosibirsk, August 7–10, 2017 - http://conf.bionet.nsc.ru/belyaev100/en). This Memorial Conference included special session on medical aspects of the genomics. In 2017, “Vavilov Journal of Selection and Breeding” published a series of memoirs publications about Prof. Belyaev (http://vavilov.elpub.ru/jour/issue/view/32/showToc).

Thematic issue of *BMC Medical Genomics* highlights the studies in medical applications of genome technologies presented at “Belyaev Readings - 2017” (BR-2017) and “High throughput sequencing in genomics” (NGS-2017) conferences in Novosibirsk (http://conf.nsc.ru/HSG2017/ru/hsg2017_hsg_thesis). Modern technologies in medicine more and more become interconnected with advances in sequencing in fundamental evolutionary studies. Previously published special issues of *BMC Evolutionary Biology* and *BMC Genomics* covered the proceedings of BGRS\SB-2016 conference and SBB-2015 School in Novosibirsk [1–4] as well as BGRS\SB-2014 event (https://bmcgenomics.biomedcentral.com/articles/supplements/volume-15-supplement-12). The materials on evolutionary biology and genetics were recently published in *BMC Evol Biol* (https://bmcevolbiol.biomedcentral.com/articles/supplements/volume-17-supplement-2) and *BMC Genetics* Supplements (https://bmcgenet.biomedcentral.com/articles/supplements/volume-18-supplement-1), correspondingly [5, 6].

This issue collected works on sequencing, genotyping, computational analysis and gene network reconstruction in human diseases.

Anastasiya Snezhkina et al. [7] describe exome analysis of carotid body tumors, rare neoplasms of the paraganglia located at the bifurcation of carotid arteries. Exome analysis of 52 carotid body tumor samples for the first time revealed the average mutation load for these tumors and also identified potential driver mutations.

The work by Maxim Ivanov and colleagues [8] discusses the results of using sequencing for ascertainment of genotypes in large group of patients with cystic fibrosis. Authors show that the choice of bioinformatics pipeline plays a crucial role in detecting clinically significant variants and highly influences diagnostic yield.

Yu-Feng Huang et al. [9] continue the theme of cancer research by describing novel way to detect DNA sequence variants in microbial cell-free DNA present in blood. The authors found that the presence of DNA from certain bacterial genera may serve as a predictive biomarker of breast carcinoma outcome.

Ulyana Boyarskikh and colleagues [10] present the results of computational analysis of the genes implicated in the response of lung cancer to certain types of the treatment. In cancer cells, small molecule Nutlin-3 reactivates p53 by interacting with the complex between p53 and its repressor Mdm-2 and causing an increase in cancer cell apoptosis. Using artificial intelligence approach embed in original software, the authors identified a set of transcription factors cooperatively binding to the promoters of genes up-regulated in the Nutlin-3 insensitive cell lines and showed that these cell lines are highly sensitive to the dual PI3K/mTOR inhibitors.

Olga Saik et al. [11] showed that associative gene networks analysis aids in dissection of the molecular underpinnings of asthma and hypertension.

Galatenko and colleagues [12] identified the prognostic value for the combinations of expression levels of laminin-encoding genes in colorectal cancer. Importantly, predictive classifiers based on the triples of laminin genes suggest an increased permeability of basal membrane in patients with higher risk of colorectal cancer recurrence.

Abeer Fadda et al. [13] discuss fundamental problem of circadian clock. The authors re-analyzed a number of mouse circadian gene expression data available from public sources. Improved methodology for data mining allowed for the discovery of functions and biological pathways in groups of genes with synchronized peak expression time. In particular, such functions as oxidative phase of energy metabolism, DNA repair, mRNA processing, lipid biosynthesis and others are separated in time.

Andrey Marakhonov et al. [14] described a case of primary, or congenital, microcephaly from the Karachay-Cherkess Republic, which was initially diagnosed with Seckel syndrome. Clinical exome sequencing of the proband revealed a novel homozygous single nucleotide deletion in *ASPM* gene. The work represents an additional support for the clinical continuum between Seckel Syndrome and primary microcephaly.

Follow-on series of related works in the areas of genomics, genetics, and plant biology discussed at “Belyaev conference – 2017” and other related meetings in Novosibirsk are published in the Special Issues of *BMC Evolutionary Biology (*https://bmcevolbiol.biomedcentral.com/articles/supplements/volume-17-supplement-2)*, BMC Plant Biology* (https://bmcplantbiol.biomedcentral.com/articles/supplements/volume-17-supplement-2)*, BMC Genetics* (https://bmcgenet.biomedcentral.com/articles/supplements/volume-18-supplement-1) (published at the end of 2017), *BMC Genomics* (https://bmcgenomics.biomedcentral.com/articles/supplements/volume-19-supplement-3), *BMC Structural Biology* (https://bmcstructbiol.biomedcentral.com/articles/supplements/volume-18-supplement-1) and *BMC Neuroscience* (https://bmcneurosci.biomedcentral.com/articles/supplements/volume-19-supplement-1) (published in parallel to this issue in 2018)*.* The Proceedings of the conference are available at http://conf.bionet.nsc.ru/belyaev100/en http://conf.bionet.nsc.ru/belyaev100/wp-content/uploads/sites/14/2017/01/BELYAEV_conf_2_08_2017.pdf.

The readers are welcome to visit Novosibirsk at the time of next XI-th BGRS\SB-2018 conference on August 20-28th in 2018 (http://conf.bionet.nsc.ru/bgrssb2018/en).
